# Pattern Synthesis Design of Linear Array Antenna with Unequal Spacing Based on Improved Dandelion Optimization Algorithm

**DOI:** 10.3390/s25030861

**Published:** 2025-01-31

**Authors:** Jianhui Li, Yan Liu, Wanru Zhao, Tianning Zhu, Zhuo Chen, Anyong Liu, Yibo Wang

**Affiliations:** School of Physics and Electronic Information, Yunnan Normal University, Kunming 650500, China; ljhkybs2022@163.com (J.L.); zhaowanru316@126.com (W.Z.); zhutn_oceancurrent@163.com (T.Z.); cz15656171360@163.com (Z.C.); 17587074972@163.com (A.L.); 17387599197@163.com (Y.W.)

**Keywords:** CENDO algorithm, logistic–tent chaotic mapping, synthesis of unequally spaced linear arrays, nonlinear control factor

## Abstract

With the rapid development of radio technology and its widespread application in the military field, the electromagnetic environment in which radar communication operates is becoming increasingly complex. Among them, human radio interference makes radar countermeasures increasingly fierce. This requires radar systems to have strong capabilities in resisting electronic interference, anti-radiation missiles, and radar detection. However, array antennas are one of the effective means to solve these problems. In recent years, array antennas have been extensively utilized in various fields, including radar, sonar, and wireless communication. Many evolutionary algorithms have been employed to optimize the size and phase of array elements, as well as adjust the spacing between them, to achieve the desired antenna pattern. The main objective is to enhance useful signals while suppressing interference signals. In this paper, we introduce the dandelion optimization (DO) algorithm, a newly developed swarm intelligence optimization algorithm that simulates the growth and reproduction of natural dandelions. To address the issues of low precision and slow convergence of the DO algorithm, we propose an improved version called the chaos exchange nonlinear dandelion optimization (CENDO) algorithm. The CENDO algorithm aims to optimize the spacing of antenna array elements in order to achieve a low sidelobe level (SLL) and deep nulls antenna pattern. In order to test the performance of the CENDO algorithm in solving the problem of comprehensive optimization of non-equidistant antenna array patterns, five experimental simulation examples are conducted. In Experiment Simulation Example 1, Experiment Simulation Example 2, and Experiment Simulation Example 3, the optimization objective is to reduce the SLL of non-equidistant arrays. The CENDO algorithm is compared with DO, particle swarm optimization (PSO), the quadratic penalty function method (QPM), based on hybrid particle swarm optimization and the gravity search algorithm (PSOGSA), the whale optimization algorithm (WOA), the grasshopper optimization algorithm (GOA), the sparrow search algorithm (SSA), the multi-objective sparrow search optimization algorithm (MSSA), the runner-root algorithm (RRA), and the cat swarm optimization (CSO) algorithms. In the three examples above, the SLLs obtained using the CENDO algorithm optimization are all the lowest. The above three examples all demonstrate that the improved CENDO algorithm performs better in reducing the SLL of non-equidistant antenna arrays. In Experiment Simulation Example 4 and In Experiment Simulation Example 5, the optimization objective is to reduce the SLL of a non-uniform array and generate some deep nulls in a specified direction. The CENDO algorithm is compared with the DO algorithm, PSO algorithm, CSO algorithm, pelican optimization algorithm (POA), and grey wolf optimizer (GWO) algorithm. In the two examples above, optimizing the antenna array using the CENDO algorithm not only results in the lowest SLL but also in the deepest zeros. The above examples both demonstrate that the improved CENDO algorithm has better optimization performance in simultaneously reducing the SLL of non-equidistant antenna arrays and reducing the null depth problem. In summary, the simulation results of five experiments show that the CENDO algorithm has better optimization ability in the comprehensive optimization problem of non-equidistant antenna array patterns than all the algorithms compared above. Therefore, it can be regarded as a strong candidate to solve problems in the field of electromagnetism.

## 1. Introduction

In radar and communication systems in engineering, there are significant differences in the requirements for antenna pattern indicators under different application requirements. For example, in some systems, to suppress the influence of the sidelobe area, the radiation energy of the main lobe is concentrated, and the directional pattern of ultra-low sidelobes is desired. In some systems, in order to resist external interference, it is desired to obtain a directional pattern that generates a specific direction of zeros. The massive multiple input multiple output (MIMO) antenna array has become one of the core technologies of the fifth generation communication system (5G) [1,2,3]. It has the advantages of improving system capacity, user experience rate, enhancing full dimensional coverage, and saving energy consumption. Therefore, in order to reduce the interference of clutter on useful signals, it is necessary to optimize the MIMO antenna array to ensure efficient and high-quality signal transmission [4]. Due to the beam scanning ability and flexible pattern formation ability of array antennas, studying the synthesis of array antenna directional patterns has important theoretical value and practical significance. As we all know, the design and synthesis of array antennas is one of the challenging problems in the field of electromagnetism, and array antenna design determines the performance of wireless systems. The synthesis of array antennas involves determining the appropriate element excitation and element position to achieve the desired antenna pattern. Linear array antennas (LAAs) can be classified into two types: uniformly spaced arrays and non-uniformly spaced arrays.

In recent years, with the study of linear array antenna pattern synthesis, more and more traditional methods, including Taylor’s method [5] and Chebyshev’s method [6], are no longer able to solve multidimensional nonlinear optimization problems. Therefore, in the research of non-uniform linear array antennas, a global optimization method is introduced; that is, new evolutionary intelligent optimization algorithms are used to solve nonlinear optimization problems, and these algorithms can obtain a low peak sidelobe level (SLL). A few well-known global optimization algorithms have been applied to the design of linear array antennas, including tabu search (TS) [7]. To minimize sidelobe level and null placements, element positions of the linear array have been optimized using the PSO algorithm [8,9,10]. For the purpose of obtaining linear antennas with low SLL and null placements in desirable directions, biogeography-based optimization (BBO) has been used [11,12]. Through optimized element spacing, the artificial bee colony (ABC) algorithm has been employed to lower the side lobe level of uniformly excited linear antenna arrays [13]. A genetic algorithm (GA) is proposed to reduce the side lobe level of a low-profile multi-subarray antenna [14]. The design of LAA has been effectively implemented using the modified spider monkey optimization (MSMO) algorithm [15], the comprehensive learning particle swarm optimization (CLPSO) algorithm [16], the chaotic particle swarm optimization (CPSO) algorithm [17], the ant lion optimization (ALO) algorithm [18], and the cat swarm optimization (CSO) algorithm [19]. LAA has been optimized using the firefly algorithm (FA) and cuckoo optimization algorithm (COA) [20,21]. Linear array antennas have been designed using the flower pollination algorithm (FPA) [22], invasive weed optimization (IWO) algorithm [23], and enhanced firefly algorithm (EFA) [24]. Nonuniform LAA has been synthesized using ant colony optimization (ACO) algorithm [25]. As a result, it is clear that the development of the electromagnetic antenna area is significantly influenced by the introduction and use of new evolutionary intelligence optimization algorithms. However, these methods, like the original DO algorithm, have certain drawbacks when looking for global solutions; for instance, early convergence to local minima may occur. To avoid such situations, using the proposed CENDO algorithm to design and optimize non-equidistant array antennas in this work will yield better results. The research objectives of this paper are firstly to reduce the SLL size of non-equally spaced antenna arrays and secondly, while reducing the array SLL size, it is necessary to generate a number of zeros in a particular direction and minimize the depth of the zeros. Meanwhile, comparing the optimization results of this new method with the original DO algorithm and other algorithms better highlights the superiority of the CENDO algorithm in solving the optimization problem of this type of non-equally spaced antenna arrays.

In this article, the DO algorithm and CENDO algorithm are introduced in detail in the Section 2. In Section 3, various benchmark functions were tested, and the performance of the CENDO algorithm was assessed by contrasting it with the DO algorithm and various other algorithms, accordingly. Then, in Section 4, a mathematical model and optimization example of the array antenna are presented, using the CENDO algorithm to optimize the element spacing of non-equidistant arrays. Compare the results with other algorithms to demonstrate the effectiveness of the CENDO algorithm in synthesizing linear array antennas. Finally, in Section 5, a summary is provided.

## 2. An Improved Version of Dandelion Optimization Algorithm

**Figure 1:**
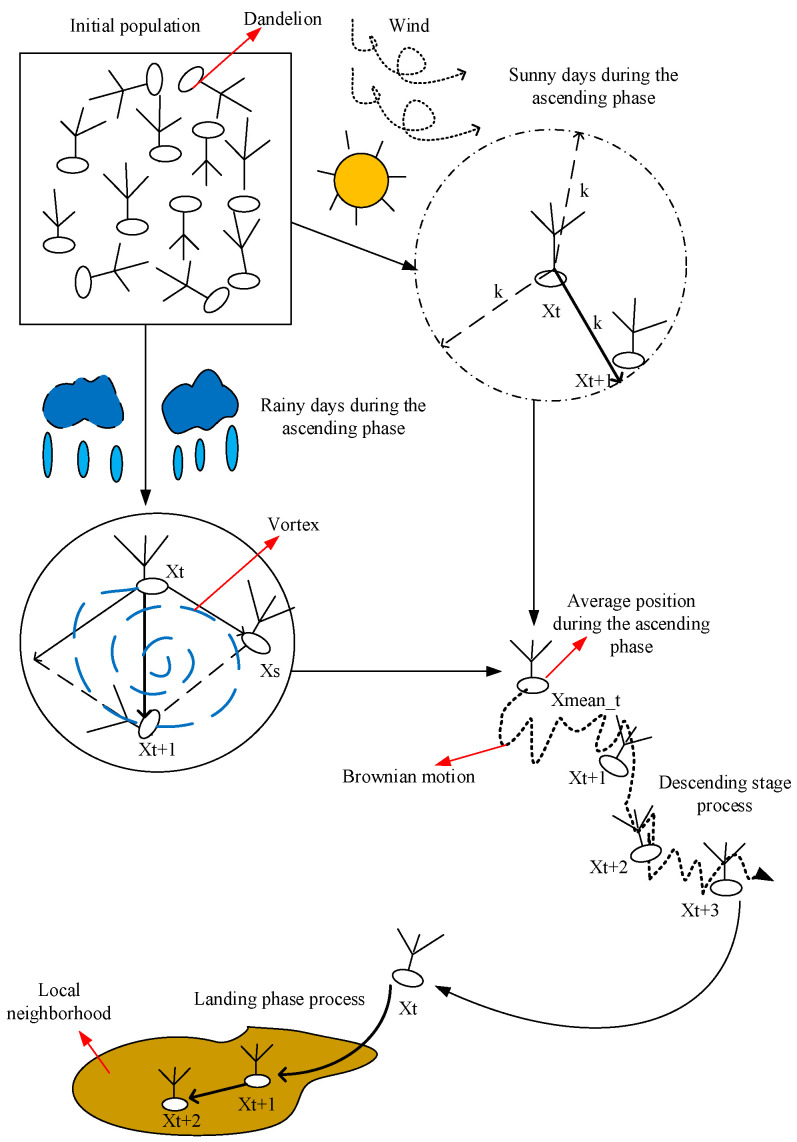
The iterative model diagram of the CENDO algorithm.

The dandelion optimization algorithm is a relatively new, nature-inspired meta-heuristic algorithm that is used to solve nonlinear global optimization problems. This section describes the dandelion optimization algorithm and the CENDO algorithm. Figure 1 vividly shows the model diagram of the CENDO algorithm.

### 2.1. The Dandelion Optimization Algorithm

In the initialization stage, the mathematical expression for the dandelion population is as follows:

(1) $$\mathrm{population}=\left[\begin{array}{ccc} {x_{1}}^{1} & \ldots & {x_{1}}^{D}\\ \ldots & \ldots & \ldots \\ {x_{N}}^{1} & \ldots & {x_{N}}^{D} \end{array}\right]$$

where *N* represents the population size and *D* represents the dimension of the variable.

Between the defined problem’s upper bound (*UB*) and lower bound (*LB*), each possible solution is created at random. The expression of the *ith* individual *X_i_* is as follows:

(2) $$X_{i}=\mathrm{rand}\times \left(UB-LB\right)+LB$$

where *i* is an integer between 1 and *N* and the *rand* refers to a number between 0 and 1. *LB* and *UB* are denoted by the following:

(3) $$\begin{array}{l} LB=\left[{lb}_{1},\ldots ,lb_{D}\right]\\ UB=\left[{ub}_{1},\ldots ,ub_{D}\right] \end{array}$$

During the initialization process, the DO algorithm takes the individual with the best fitness value as the initial optimum body, which is approximated as the most suitable location for the survival and reproduction of dandelions. The mathematical expression *X_elite_* for the initial optimum body is as follows:

(4) $$\begin{array}{l} f_{best}=\min\left(f\left(X_{i}\right)\right)\\ X_{elite}=X\left(find\left(f_{best}=f\left(X_{i}\right)\right)\right) \end{array}$$

where *find* () refers to two indexes with the same values.

When dandelions are in the process of rising, the weather is categorized as sunny or rainy based on factors such as wind speed, air humidity, and other variables.

In the case of a sunny day, dandelions are randomly scattered around the search area by the wind. The higher the wind speed, the higher the dandelions fly and spread farther. In this case, the mathematical model of dandelion evolutionary iteration is as follows:

(5) $$X_{t+1}=X_{t}+\alpha \times v_{x}\times v_{y}\times \ln Y\times \left(X_{s}-X_{t}\right)$$

where *X_t_* is the location of the dandelion at iteration *t*, *X_s_* stands for the randomly selected position in the search space during iteration *t*, and Equation (6) provides the expression for the randomly created location.

(6) $$X_{s}=\mathrm{rand}\left(1,Dim\right)\times \left(UB-LB\right)+LB$$

where *lnY* denotes a lognormal distribution subject to μ = 0 and $\sigma^{2}$= 1, and its mathematical formula is as follows:

(7) $$\ln Y=\left\{\begin{array}{l} \frac{1}{y\sqrt{2\pi }}\exp\left[-\frac{1}{2\sigma^{2}}{\left(\ln y\right)}^{2}\right],y\geq 0\\ 0,y<0 \end{array}\right.$$

In Equation (7), *y* represents the standard normal distribution *N* (0, 1). The mathematical expression for *α*, an adaptive parameter used to modify the search step length, is as follows:

(8) $$\alpha =\mathrm{rand}\left(\;\right)\times \left(\frac{1}{T^{2}}t^{2}-\frac{2}{T}t+1\right)$$

In Equation (8), *α* is a random number between [0, 1] and follows the principle of nonlinear reduction. The maximum iterations are denoted by *T*. During ascent, dandelions create vortices, and *v_x_* and *v_y_* are the two parts of the force that the vortex creates. To determine *v_x_* and *v_y_*, we should use Equation (9).

(9) $$\begin{array}{l} r=\frac{1}{e^{\theta }}\\ v_{x}=r\times \cos\theta \\ v_{y}=r\times \sin\theta \end{array}$$

where *θ* is a random number between [−*π*, *π*]. *r* represents the rising vortex distance.

In the case of rainy days, dandelions cannot be carried by the wind to distant places, they can only spread in a small local area, so the mathematical expression for this stage is as follows:

(10) $$X_{t+1}=X_{t}\times k$$

where *k* determines the dandelion’s local search domain, which is determined by Equation (11).

(11) $$\begin{array}{l} q=\frac{1}{T^{2}-2T+1}t^{2}-\frac{2}{T^{2}-2T+1}t+1+\frac{1}{T^{2}-2T+1}\\ k=1-\mathrm{rand}\left(\;\right)\times q \end{array}$$

where *q* represents the factor that determines the control step size based on the number of iterations and the maximum number of iterations.

For the above two weather conditions, the iterative mathematical model for dandelion evolution is as follows:

(12) $$X_{t+1}=\left\{\begin{array}{l} X_{t}+\alpha \times v_{x}\times v_{y}\times \ln Y\times \left(X_{s}-X_{t}\right),randn<1.5\\ X_{t}\times k,else \end{array}\right.$$

where *randn* denotes a random number that adheres to the normal distribution. In both scenarios, the cut-off point is 1.5, which is more favorable for the DO algorithm’s global convergence [26].

To ensure the stability of dandelion landing, when they are in decline, the average position data from the ascending stage is utilized in detailing the iteration procedure during the descending stage. The corresponding mathematical expression for this stage is as follows:

(13) $$X_{t+1}=X_{t}-\alpha \times \beta_{t}\times \left(X_{\mathrm{mean}\_t}-\alpha \times \beta_{t}\times X_{t}\right)$$

where *β_t_* is a random number from the standard normal distribution denotes Brownian motion [27]. The mathematical expression for *X_mean_t_*, which stands for the population’s average location, is as follows:

(14) $$X_{\mathrm{mean}\_t}=\frac{1}{N}\sum_{i=1}^{N}X_{i}$$

When dandelions land, the search agent uses the current optimal body’s position information to iterate in its neighborhoods as the iteration goes on. Finally, the global optimal solution can be found. Therefore, the DO algorithm reaches the global optimal outcome. Equation (15) describes this behavior.

(15) $$X_{t+1}=X_{\mathrm{elite}}+\mathrm{levy}\left(\lambda \right)\times \alpha \times \left(X_{\mathrm{elite}}-X_{t}\times \delta \right)$$

where *X_elite_* denotes the dandelions’ ideal placement in the *ith* iteration. *Levy* (λ), which is computed using Equation (16), represents the Levy flight function [28].

(16) $$\mathrm{Levy}\left(\lambda \right)=s\times \frac{w\times \sigma }{{\left|t_{1}\right|}^{\frac{1}{\beta }}}$$

Consider the value of *β* is 1.5 in Equation (16) [26], where *s* is set at 0.01 [26], and *w* and *t*_1_ are random values between [0, 1] [26]. The mathematical expression of σ is written as follows:

(17) $$\sigma =\left(\frac{\Gamma \left(1+\beta \right)\times \sin\left(\frac{\pi \beta }{2}\right)}{\Gamma \left(\frac{1+\beta }{2}\right)\times \beta \times 2^{\left(\frac{\beta -1}{2}\right)}}\right)$$

The expression for parameter *δ* is given by Equation (18), and this parameter is a linear function that increases from [0, 2] [26].

(18) $$\delta =\frac{2t}{T}$$

In this process, the Levy flight coefficient can allow dandelions to cross to other distant locations with a greater probability. It helps the DO algorithm precisely converge to the global optimal solution.

### 2.2. Initialization of Logistic–Tent Chaotic Mapping

**Figure 2:**
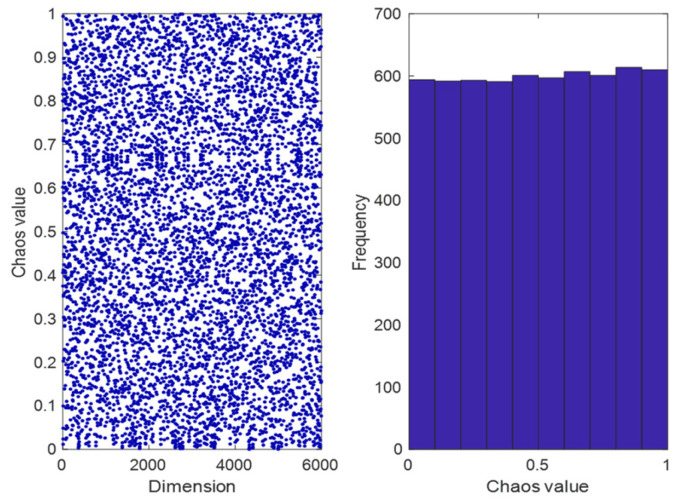
Logistic–tent chaotic mapping distribution diagram (**left**) and the mapping distribution histogram (**right**).

In this section, we propose an improved initialization method for the original dandelion optimization algorithm. We introduce the logistic–tent chaotic mapping algorithm, which generates a more uniform initial population compared to the original algorithm. In the synthesis application of array antennas, pseudo-random sequences are widely used. Among them, the mathematical definition of the logistic–tent chaotic mapping algorithm is extremely sensitive to initial values and parameters, and it can generate a large number of chaotic sequences with good randomness, which provides a good research model for generating high-quality initial populations [29]. Therefore, this article replaces traditional pseudo-random sequences with pseudo-random sequences generated by the logical–tent chaotic mapping algorithm, laying a good foundation for subsequent algorithm iterations. The formula for the chaotic mapping of the logical tent is given in Equation (19). The mapping distribution diagram and the mapping distribution histogram are shown in Figure 2.

(19) $$X_{n+1}=\left\{\begin{array}{l} \left[rx_{n}\left(1-x_{n}\right)+\frac{\left(4-r\right)}{2}x_{n}\right]\mathrm{mod}1,if\;x_{n}<0.5\\ \left[rx_{n}\left(1-x_{n}\right)+\frac{\left(4-r\right)\left(1-x_{n}\right)}{2}\right]\mathrm{mod}1,if\;x_{n}\geq 0.5 \end{array}\right.$$

where *X_n+*1*_* is system variable; *r* is control parameter, set to 0.1; and $x_{n}\in \left[0,1\right]$.

### 2.3. Iterative Exchange of Dandelion Ascending Process

**Figure 3:**
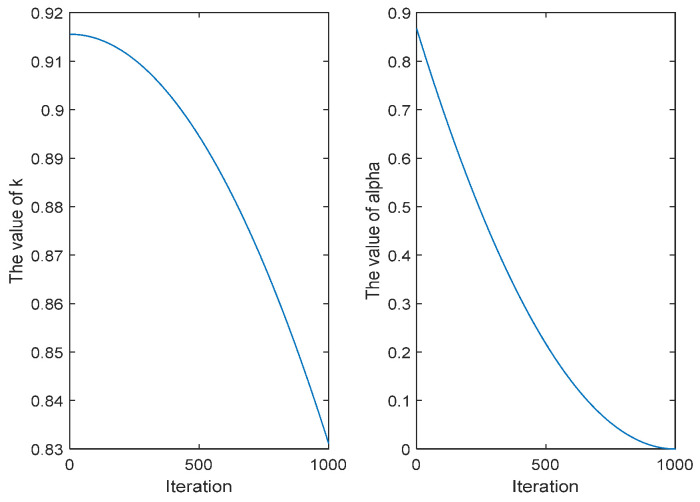
Plots of changes in parameter *k* and parameter *α*.

In the CENDO algorithm, the iterative models of sunny days and rainy days in the rising stage are exchanged, because in the original DO algorithm, dandelion needs to fly to a farther search area under the action of wind to seek optimization in the case of sunny days in the rising stage, but the step size parameter of the iterative formula in this case, *α* value, is too small, and the search area is very small during exploration, which leads to a small range of dandelion to find the most suitable location for growth, which is easy to fall into a local optimum. In the rainy and rainy days during the ascending stage, dandelion only needs to seek optimization in its nearby small neighborhood, and in this case, the step parameter *k* value of the iterative formula is relatively large. During the development of the neighborhood stage, the search area is too large, making the algorithm convergence speed slow and unable to find the optimal solution. The iterative formula of the rising stage in the CENDO algorithm is given by Equation (20), where the changes of parameter *k* and parameter *α* are shown in Figure 3.

(20) $${X_{t+1}}^{\prime }=\left\{\begin{array}{l} X_{t}\times k,randn<1.5\\ X_{t}+\alpha \times v_{x}\times v_{y}\times \ln Y\times \left(X_{s}-X_{t}\right),\mathrm{else} \end{array}\right.$$

Therefore, the iteration formula in the ascending stage is exchanged, and then the step parameters *α* and *k* in the case of sunny and rainy days are adjusted so that while iterating on sunny days, the step size is longer and the search area range is larger, whereas iterating on rainy days results in a smaller search area range, a smaller step size, and a stronger local search capability. This improvement not only improves the ability of global search and local search but also avoids the algorithm falling into local optimization and accelerates algorithm convergence.

### 2.4. The Descending Process of Dandelion

The descent stage of the CENDO algorithm is the same as the descent stage of the original DO algorithm, which is based on the average position information of the rising stage of dandelion for iterative optimization in this stage. The iterative mathematical model of dandelion in this stage is expressed by Equation (13).

From the decline process of dandelion, the average position information of the population is very important, and it is very significant to determine the evolution direction of individual iterative updates. In the iterative updating process, Brownian motion helps dandelion to avoid falling into local extremum when searching for optimization and makes the population settle in the region close to the global optimal solution.

### 2.5. Nonlinear Control Factor of Dandelion Landing Process

In the iteration of the landing phase of dandelion, the linear growth factor δ in Equation (18) is changed to the exponential nonlinear growth factor $\delta^{\prime }$, so as to better control the position of dandelion *X_t+*1*_*. Because in the original DO algorithm, this stage searched for the optimal solution in the local neighborhood, using a linear growth factor that increased the search step quickly, resulting in crossing the optimal solution, while the improved nonlinear factor grew slowly. In this way, it can avoid that the dandelion position crosses the optimal value due to the large step size in the early stage of development and can increase the search weight so that the algorithm can quickly converge to the global optimal solution in the later stage, realizing the dynamic control and balance of the algorithm in the entire convergence process. The modified growth factor $\delta^{\prime }$ is given by Equation (21) and incorporated into the CENDO algorithm.

(21) $$\delta^{\prime }=\log\left(4\times \left(\exp\left(2t^{2}/T^{2}\right)/4\right)\right)$$

**Figure 4:**
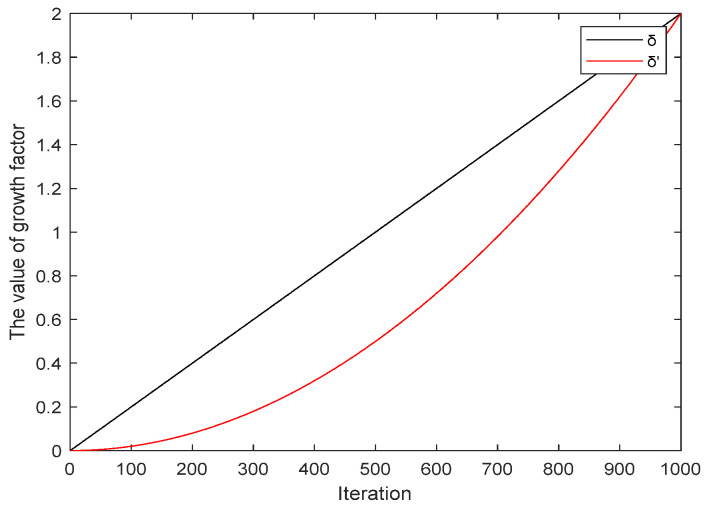
Comparison graph of growth factors between the original DO algorithm and the CENDO algorithm.

A comparison of the values of the linear growth factor δ in the original DO algorithm with the values of the nonlinear growth factor $\delta^{\prime }$ in the CENDO algorithm is shown in Figure 4.

Obviously, the value of $\delta^{\prime }$ in the CENDO algorithm grows slowly during the iteration process, which is beneficial for the algorithm to slowly find the optimal value in nearby neighborhoods with a more accurate step size and can enable the algorithm to quickly converge to the global optimal solution in the later stage, thus achieving dynamic control and balance throughout the entire convergence process.

## 3. Benchmark Function Testing

**Figure 5:**
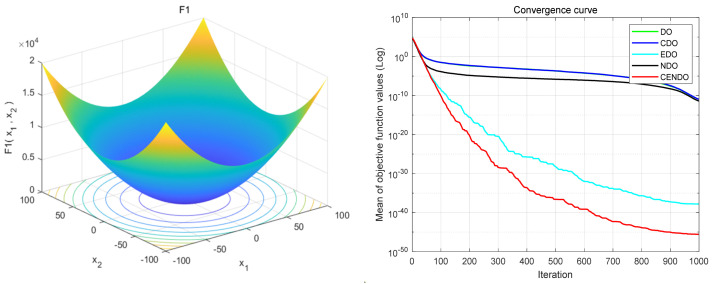

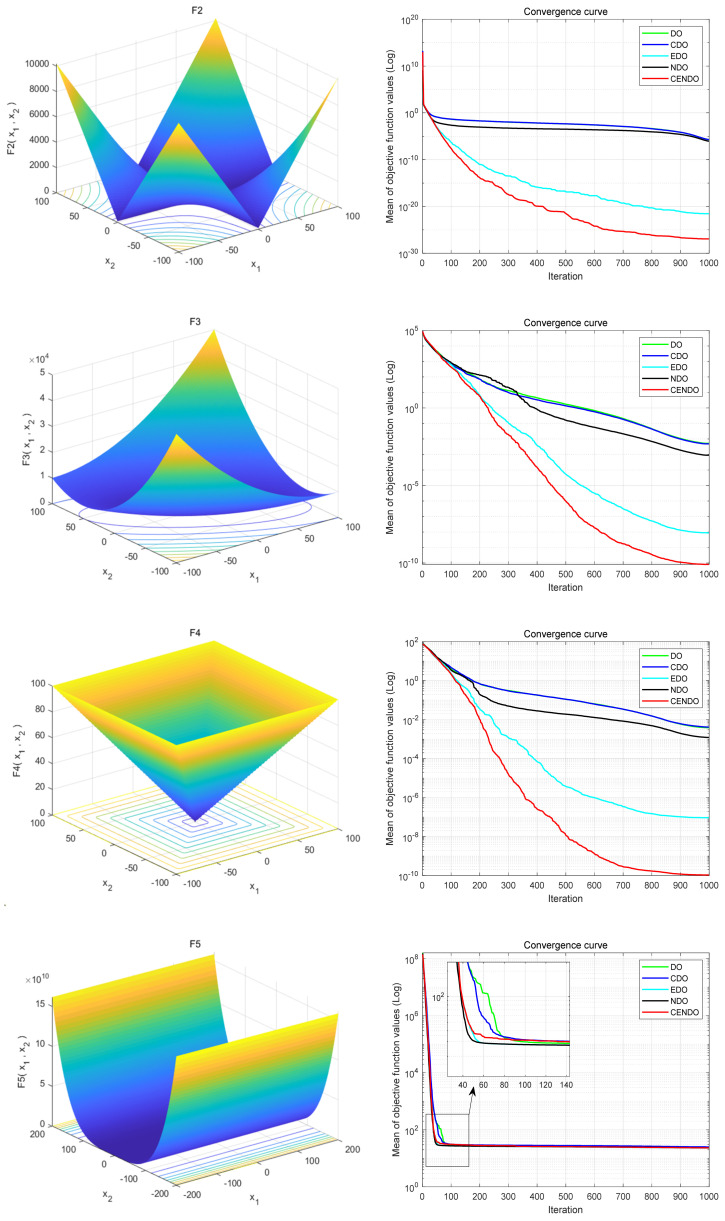

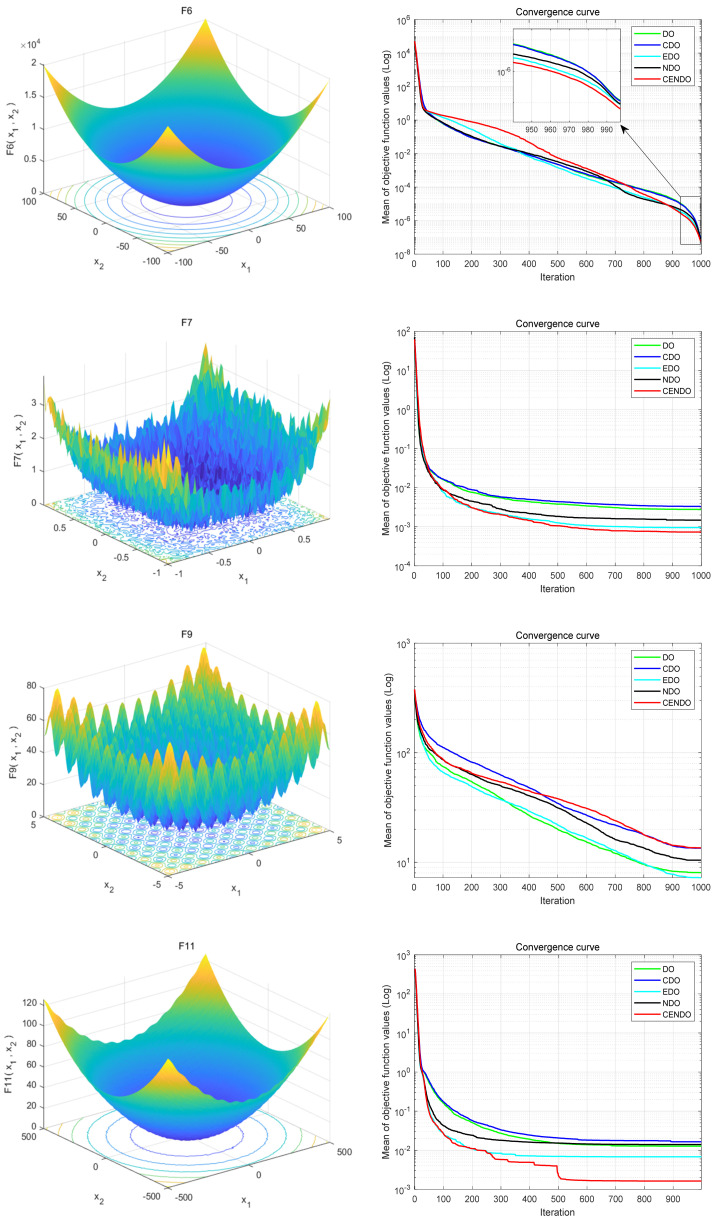

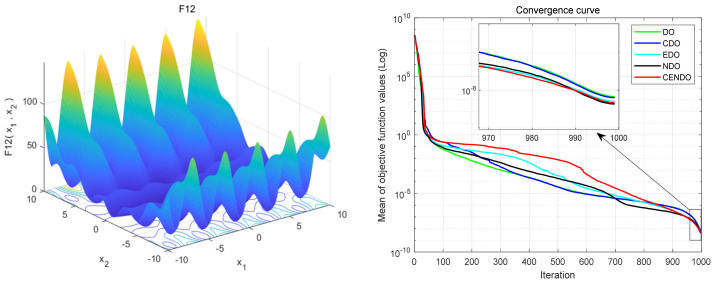
The 3D three-dimensional images (**left**) and comparison plots of iterative curves (**right**).

The first part of this section introduces the benchmark functions used. Table 1 shows a comprehensive explanation of these test functions. The second part mainly verifies the effectiveness of the CENDO algorithm by using multiple well-known benchmark functions from the following four aspects: Firstly, only adding logical tent chaotic mapping improvement points separately (abbreviated as the CDO algorithm); secondly, only exchanging improvement points of iterative formulas separately (abbreviated as the EDO algorithm); thirdly, only changing the improvement points of the growth factor separately (abbreviated as the NDO algorithm); and fourth, to combine the three improvement points to form the CENDO algorithm proposed in this article. The comparison of iteration curves for test functions with different improvement points and their matching 3D stereo graphics are shown in Figure 5. Table 2 shows the test results; among them, bold data represents better values than the DO algorithm. The third part compares the test function results of the CENDO algorithm with the PSO algorithm, the gravity search algorithm (GSA), and PSOGSA, further proving the effectiveness of the CENDO algorithm in optimization problems. The test results are presented in Table 3, where the data in bold indicates the best results.

### 3.1. Introduction to Test Functions

Below, a brief introduction will be given to these test functions. F_1_–F_7_ is the unimodal test function section, and F_9_, F_11_, and F_12_ are the multimodal test function sections. Among them, F_1_ is a continuous, concave, unimodal, and variable separable function; F_5_ is a unimodal, variable non-separable function, with a very narrow concave valley between local and global optima; F_11_ is a multimodal, variable separable function with many local optimal solutions; F_11_ is a multimodal, variable inseparable function. All the above functions obtain a global optimal solution of 0 at *X* = 0.

### 3.2. Test Function Results of CENDO Algorithm and DO Algorithm

The population size and the maximum number of iterations are both set to the same values. They are both set to 100 and 1000, respectively. Each of the two algorithms is run 50 times separately.

According to the comparison graph of the iteration curve, it can be analyzed that in multiple test functions, F_1_–F_4_, F_6_, F_7_, and F_11_, the CENDO algorithm exhibits faster convergence performance compared to the DO algorithm, CDO algorithm, EDO algorithm, and NDO algorithm, while also obtaining smaller mean values. This also proves that the performance of the CENDO algorithm is more stable. Therefore, the CENDO algorithm will be better and more stable in the study of optimization problems. By comparing the results of Table 2, it was found that the results obtained by using three separate improvement points were better than those obtained by the DO algorithm in most test functions, and the results obtained by the CENDO algorithm were smaller than those obtained by the DO algorithm in terms of best value, worst value, mean value, and standard deviation (SD). Except for the worst value, mean value, and SD in the F_9_ function, these three results were not as good as the DO algorithm, but the difference was not significant. Moreover, combining the three improvement points together yields better results than a single improvement point, which proves that the CENDO algorithm has better optimization performance than the original DO algorithm. Because these test functions are often used as metrics to evaluate the performance of optimization algorithms. So, the experimental results obtained are relatively accurate.

### 3.3. Test Function Results of CENDO Algorithm and Other Algorithms

A comparison of three well-known algorithms, namely PSO, GSA, and PSOGSA, was made to examine the performance of the CENDO algorithm. The population size and maximum number of iterations for each of the four algorithms were, fairly, set to 30 and 1000, respectively [30]. All algorithms are run 30 times independently in order to gather statistical findings.

As can be seen from Table 3, for the best value of 30 runs on these test functions mentioned above, the CENDO algorithm is the best on eight functions, PSO is the best on one function, PSOGSA is the best on one function, and GSA is the best on no function. For the mean value of 30 runs on 10 test functions, the CENDO algorithm performs the best on 7 functions, and PSOGSA is the best on 3 functions. PSO and GSA are the best on no function. Therefore, the number of functions that perform better using the CENDO algorithm is close to twice to three times that of PSOGSA and PSO. Statistically speaking, the CENDO algorithm shows a higher performance on these test functions. It can be broadly concluded that the CENDO algorithm is more likely to be the best choice for optimizing nonlinear and high-dimensional problems.

## 4. Sparse Array

This section describes the mathematical model of a non-equally spaced linear array and various examples of antenna model simulations and analyses the experimental results.

### 4.1. Array Geometry and the Array Factor

In the past few years, many scholars have conducted research concerning unequally spaced linear array antennas [13]. Compared with equally spaced linear array antennas, it is not only able to reduce the complexity and cost of the feed network but also enhance the gain and directionality of the antenna.

**Figure 6:**
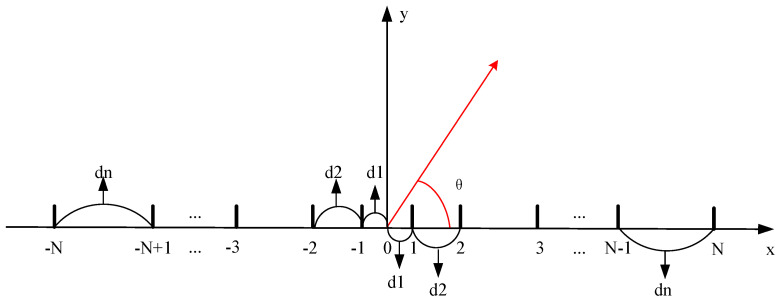
Illustration of the geometry of a linear array antenna (*d_i_* denotes array element spacing).

Figure 6 depicts a general linear array geometry with 2*N* array elements placed on the *x*-axis.

The equation for the array factor of an equidistant linear array antenna is as follows:

(22) $$AF\left(\theta \right)=2\sum_{n=1}^{N}I_{n}\cos\left({kx}_{n}\cos\left(\theta \right)+\varphi_{n}\right)$$

where *I_n_*, *φ_n_*, and *x_n_* are the excitation amplitude, phase, and position of the *nth* element in the array; *k* is the wave number and is given by 2π/λ; and *θ* is the pitch angle.

The non-equally spaced array antenna is optimized for the distance between adjacent array elements, which is given a constant excitation, i.e., excitation current *I_n_* = 1 and excitation phase *φ_n_* = 0. Therefore, the array factor (*AF*) of the symmetric array antenna is given by Equation (23), written as follows:

(23) $$AF(\theta )=2\sum_{n=1}^{N}\cos(kx_{n}\cos(\theta ))$$

When the number of array elements is an odd number of (2*N* + 1), the calculation of the array factor is changed accordingly by adding 1 to Equation (23), i.e., the array factor is adjusted to Equation (24).

(24) $$AF(\theta {)}^{\prime }=2\sum_{n=1}^{N}\cos(kx_{n}\cos(\theta ))+1$$

The placement of each antenna in a linear array antenna is very important to reduce the SLL and produce a high-gain antenna directional pattern. If each antenna is placed too close together, this can lead to mutual coupling effects, and conversely, placed too far apart, this can lead to the appearance of grating lobes. Therefore, the conditions to be satisfied for optimum antenna placement are given by Equation (25).

(25) $$\begin{array}{l} \left|x_{i}-x_{j}\right|>0.25\lambda \\ i=1,2,\ldots ,N,i\neq j \end{array}$$

where *x_i_* and *x_j_* represent the positions of any two different array elements in the array antenna.

The formula for the directivity (*D_LAA_*) of a symmetric linear array with unequal spacing is given by Equation (26).

(26) $$D_{LAA}=10\log\left(\frac{4\pi }{\int_{0}^{2\pi }\int_{0}^{\pi }f^{2}\left(\theta ,\varphi \right)\sin\theta d\theta d\varphi }\right)$$

where *θ* and *φ* are the pitch angle and the azimuth angle, but in linear array pattern synthesis, only *θ* is an angular variable and *φ* does not need to be considered, and *f* (*θ*, *φ*) is the normalized direction function of the antenna in the maximum radiation direction.

### 4.2. Experimental Simulation Results of Optimizing Array Element Spacing

This part uses the CENDO algorithm for optimizing the position of a linear array antenna to obtain the desired pattern through experimental simulations. A comparison with other algorithms is also made to illustrate the effectiveness of the CENDO algorithm.

#### 4.2.1. Minimize Antenna Sidelobe Level

As shown in [9,11], the fitness function used to reduce peak SLL is written as follows:

(27) $$\mathrm{Fitness}=\min\left(\max\left(20\log\left|AF\left(\theta \right)\right|\right)\right)$$

Here, $\max\left(20\log\left|AF\left(\theta \right)\right|\right)$ gives the peak SLL and $AF\left(\theta \right)$ is the array factor given by Equation (23).

In order to reduce the peak SLL, this subsection provides three examples of experimental simulations that demonstrate how the CENDO algorithm can be used to optimize the spacing of the antenna array elements. For these three cases, the fitness function provided by Equation (27) is applied.

**Figure 7:**
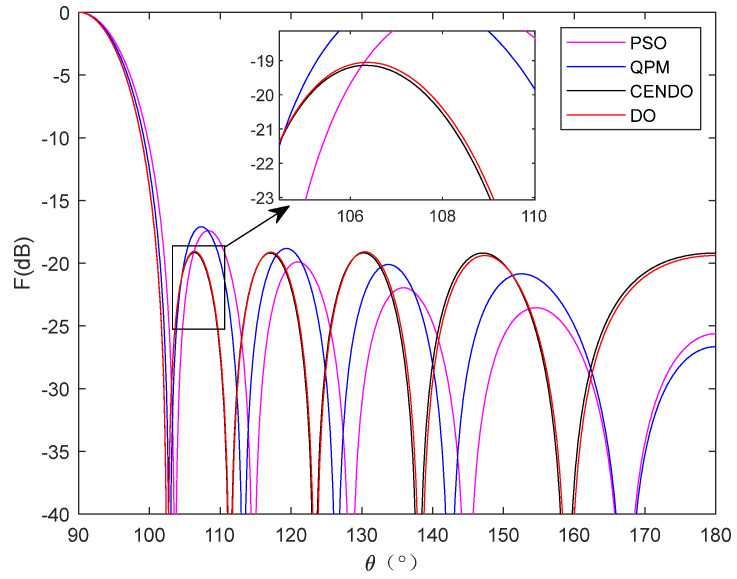
Radiation patterns of optimized 10-element arrays by four algorithms.

**Figure 8:**
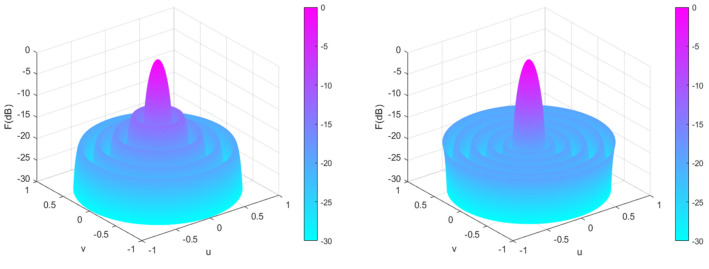
Unoptimized 3D radiograms (**left**) and 3D radiograms optimized by the CENDO algorithm (**right**).

Experimental Simulation 1: We consider a 2*N* = 10 linear array to achieve the lowest possible SLL in regions *θ* = [0°, 78°] and *θ* = [102°, 180°]. The population size and the maximum number of iterations are set to 20 and 400, respectively. Table 4 shows the peak SLL values, main lobe width, and directivity obtained by PSO [31], QPM [31], DO, and CENDO algorithms, as well as the positions of the array elements obtained by optimizing. Radiation patterns of the four optimized arrays using different algorithms are plotted in Figure 7. From Figure 7, it can be intuitively seen that each algorithm optimizes the directional pattern results of the array. As it is a symmetrical array, only the range of 90°–180° was selected for observation. From the figure, it can be seen that the areas outside the main lobe are all side lobes. The SLL optimized by the CENDO algorithm is lower, so its ability to reduce the SLL of the array is better than other compared algorithms. The 3D radiograms (two-dimensional planes rotating 180° around the z-axis) before and after optimization are shown in Figure 8. The peak SLL obtained by the CENDO algorithm is −19.14 dB, which is 1.73 dB and 2.05 dB lower than the peak SLL of the PSO [31] and the QPM [31] algorithm-optimized arrays. The peak SLL decreases from −19.05 dB to −19.14 dB (by 0.09 dB) compared with the DO algorithm. In terms of main lobe width, the main lobe width optimized by CENDO and DO algorithms is narrower than that optimized by PSO [31] and QPM [31] algorithms. Moreover, compared with the original DO algorithm, CENDO algorithm achieves lower sidelobe levels without broadening the main lobe. Based on the above, the CENDO algorithm is more effective in reducing antenna sidelobe levels. In addition, for directivity, the directivities obtained using other algorithms are 9.7306 dB, 9.9120 dB, 10.0871 dB, and 10.0930 dB, respectively. However, the directivity obtained using CENDO algorithm are larger than theirs, indicating that the energy radiated by the array antenna is more concentrated, and this also shows that CENDO algorithm is superior to other algorithms in terms of effectiveness.

Experimental Simulation 2: This example uses the CENDO algorithm to optimize a 2*N* = 16 linear array antenna, with the aim of reducing peak sidelobe level. The aim is to suppress the maximum SLL in the regions *θ* = [0°, 82°] and *θ* = [98°, 180°]. The population size is 30, and the number of iterations for each run is set to 500. Unlike the previous example, this model fixes the position of the first element and the position of the last element. The calculation formula is Equation (28).

(28) $$\left\{\begin{array}{l} x_{1}=0.25\lambda \\ x_{N}=\frac{\left(2N-1\right)d}{2} \end{array}\right.$$

where the uniform LAA’s standard spacing is indicated by *d* = 0.5λ.

**Figure 9:**
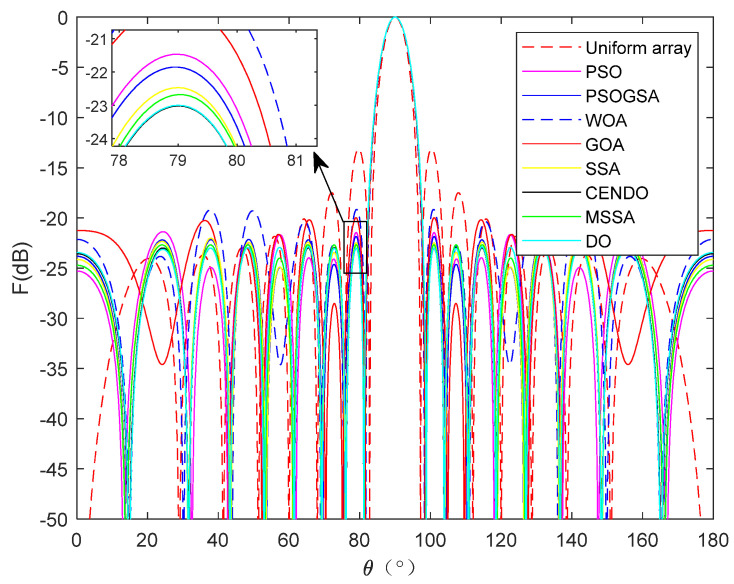
16-element linear array radiation pattern optimized using different algorithms.

**Figure 10:**
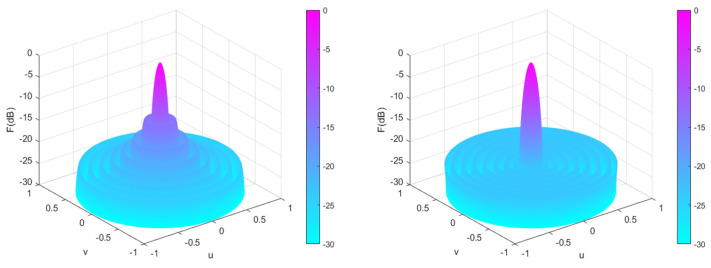
Unoptimized 3D radiograms (**left**) and 3D radiograms optimized by the CENDO algorithm (**right**).

Eight algorithms, PSO [32], PSOGSA [32], WOA [32], GOA [32], SSA [32], MSSA [32], DO, and CENDO, are used to optimize the position of the array. The array pattern optimized by these algorithms is given in Figure 9. From Figure 9, it can be intuitively seen that each algorithm optimizes the directional pattern results of the array. It is not difficult to see that the areas outside the main lobe are all side lobes. The SLL optimized by the CENDO algorithm is lower, so its ability to reduce the SLL of the array is better than other compared algorithms. Figure 10 shows the 3D radiograms (two-dimensional planes rotating 180° around the z-axis) before and after optimization. Table 5 provides a summary of the element positions, the peak SLL values, the main lobe width, and the directivity that are optimized using these nature-inspired optimization algorithms. As you can see from the table, the conventional method (uniform array), PSO [32], PSOGSA [32], WOA [32], GOA [32], SSA [32], MSSA [32], and DO algorithms optimized array provide a peak SLL of −13.1476 dB, −21.3693 dB, −21.8484 dB, −19.1546 dB, −19.9808 dB, −22.0177 dB, −22.6768 dB, and −22.8766 dB, respectively. The CENDO algorithm optimized array gives a peak SLL of −22.9377 dB, which is 9.7901 dB lower, 0.2609 dB lower, and 0.0611 dB lower as compared to the uniform array, MSSA [32]-optimized array, and DO optimized array. Moreover, the main lobe width obtained by the CENDO algorithm is not widened compared to the SSA [32], MSSA [32], and DO algorithms. Although its main lobe width is slightly wider compared to PSO [32], PSOGSA [32], WOA [32], and GOA [32] algorithms, the peak SLL obtained by the CENDO algorithm is much lower than them. From the table, it can also be seen that the directivities obtained by each algorithm are almost the same, with values of around 11.8 dB. Therefore, the concentration of electromagnetic wave energy radiated by the array antenna in the main lobe direction is equivalent.

Experimental Simulation 3: a linear array with 2*N* = 28 elements is considered to achieve the minimum SLL. The main lobe range is controlled within *θ* = [86°, 94°]. Five algorithms, PSO [19], CSO [19], RRA [33], DO, and CENDO, are used to optimize the array element positions. The population size of all algorithms is set to 40, and the maximum number of iterations is set to 1000.

**Figure 11:**
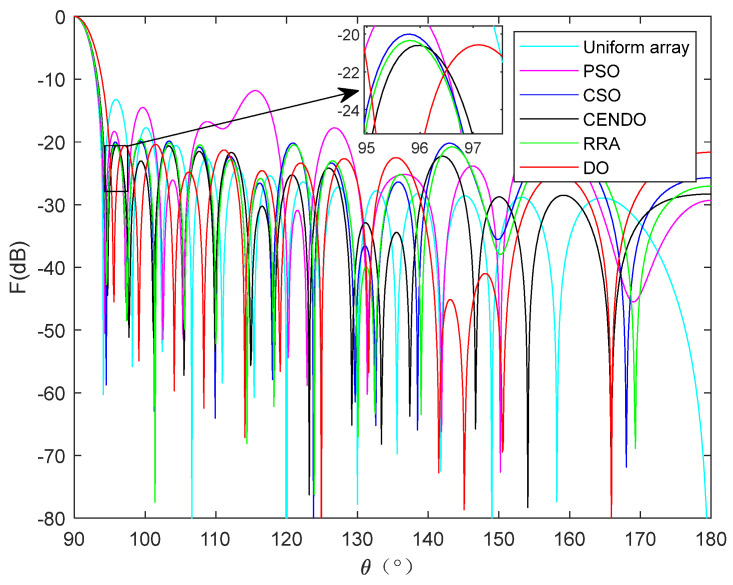
**A** 28-element linear array antenna radiation pattern.

**Figure 12:**
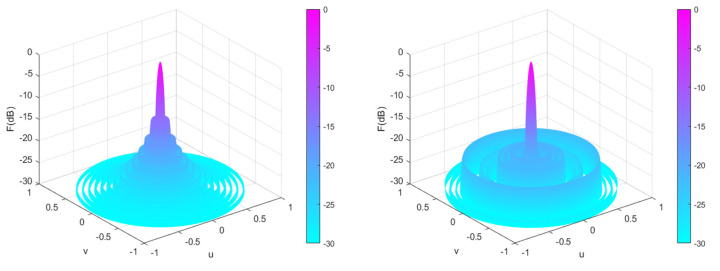
Unoptimized 3D radiograms (**left**) and 3D radiograms optimized by the CENDO algorithm (**right**).

Figure 11 shows a 28-element linear array pattern optimized using five algorithms. From Figure 11, it can be intuitively seen that each algorithm optimizes the directional pattern results of the array. As it is a symmetrical array, only the range of 90°–180° is selected for observation. From the figure, it can be seen that the areas outside the main lobe are all side lobes. The SLL optimized by the CENDO algorithm is lower, so its ability to reduce the SLL of the array is better than other compared algorithms. The 3D radiograms (two-dimensional planes rotating 180° around the z-axis) before and after optimization are shown in Figure 12. Optimized array element positions, directivity, main lobe width, and the results of peak SLL suppression have been summarized in Table 6. As can be seen in Table 6, using the CENDO algorithm to optimize the array antenna, the directivity obtained is 14.4040, which is larger than the directivity obtained using the DO, RRA [33], and CSO [19] algorithms, so it is more concentrated when the antenna radiates energy. Although it is slightly smaller than the directivity obtained by the PSO [19] algorithm, which is only about 0.2 dB lower, the peak SLL obtained by the CENDO algorithm is much lower than that obtained by the PSO [19] algorithm, at around 3 dB. It can also be seen that the peak SLL values provided by the PSO [19] algorithm, the CSO [19] algorithm, and the RRA [33] algorithm are −17.09 dB, −20.04 dB, and −20.37 dB, and the peak SLL obtained by the DO algorithm is −20.39 dB. The CENDO algorithm can reduce the peak SLL value to −20.60 dB, which is equivalent to a decrease of 0.21 dB compared to the DO algorithm. Moreover, it is 7.37 dB, 3.51 dB, 0.56 dB, and 0.23 dB lower than the uniform array, PSO [19]-, CSO [19]-, and RRA [33]-optimized arrays, respectively, although the CENDO algorithm causes a slight broadening of the main lobe compared to them. Furthermore, compared with the original DO algorithm, the CENDO algorithm not only reduces the peak SLL, but also ensures that the main lobe does not widen. So, it can be confirmed that the CENDO algorithm has better optimization performance in reducing peak sidelobe levels.

#### 4.2.2. Minimize Peak SLL and Form Deep Zeros in Specified Directions

In this section, while reducing the peak SLL, we need to achieve deep zero in the specified direction to counteract the effect of strong interference on the array performance, with the fitness function shown in the following Equation (29) [11,31]:

(29) $$\mathrm{Fitness}=\sum_{i}\frac{1}{\Delta \theta_{i}}\int_{\theta_{li}}^{\theta_{ui}}{\left|AF\left(\theta \right)\right|}^{2}d\theta +{\sum_{k}\left|AF\left(\theta_{k}\right)\right|}^{2}$$

where *θ_li_* and *θ_ui_* are the spatial regions in which SLL is suppressed and Δ*θ_i_ = θ_ui_ − θ_li_*. The null direction is given by *θ_k_*. The first term of the fitness function in Equation (29) accounts for SLL suppression, and the second term takes into consideration of the nulls in the desired directions. *AF* (*θ*) is the array factor given by Equation (23). The fitness function used for Experimental Simulation 4 and Experimental Simulation 5 are given by Equation (29).

**Figure 13:**
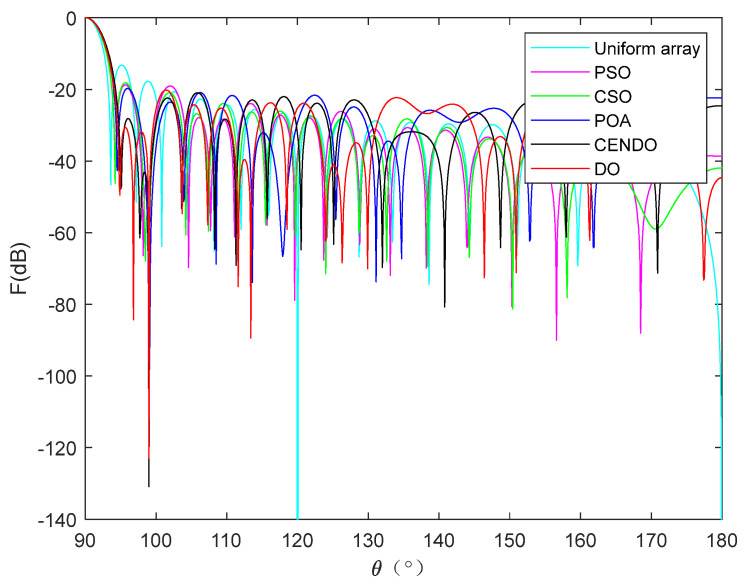
**A** 32-element linear array antenna radiation pattern.

**Figure 14:**
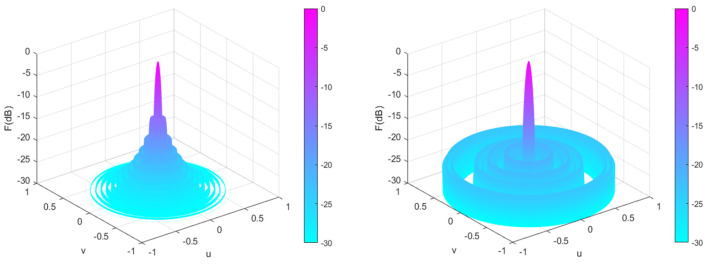
Unoptimized 3D radiograms (**left**) and 3D radiograms optimized by the CENDO algorithm (**right**).

Experimental Simulation 4: the fourth example illustrates the synthesis of a 32-element array for the minimum sidelobe levels in the regions *θ* = [0°, 85°] and *θ* = [95°, 180°], where the expected zero points are at *θ* = 81° and *θ* = 99°. 50 make up the population size, and there are 1000 iterations total. Then, in Table 7, the optimized array element positions, peak sidelobe levels, and nulls depth obtained by the CENDO algorithm and other algorithms are presented. In Table 8, the main lobe width of the CENDO algorithm and other algorithms are compared. For a more intuitive experience, the array patterns obtained by the CENDO algorithm, DO algorithm, PSO [31], CSO [19], and POA [34] are plotted in Figure 13. From Figure 13, it can be visually observed the directional pattern results of the array optimized by each algorithm. Due to its symmetrical array, only the range of 90°–180° is selected for observation. From the figure, it can be seen that the optimized SLL of the CENDO algorithm is the lowest, and its null depth in the specified direction is also the lowest. Therefore, the CENDO algorithm has a better ability to reduce array SLL and nulls compared to other comparative algorithms. Figure 14 shows the 3D radiograms (two-dimensional plane rotates 180° around z-axis) before and after optimization.

It is clear from Table 7 that the uniform array offers a null depth of −17.82 dB. The PSO [31] algorithm provides nulls depth of −62.12 dB, while the CSO [19] algorithm provides nulls depth of −80.27 dB. The POA [34] provides a null depth of −106.92 dB. DO algorithm gives −122.9 dB nulls. However, the placement of nulls up to −130.9 dB deep at the desired directions is achieved by the CENDO algorithm. It is seen that the nulls depth obtained by using the CENDO algorithm-optimized array is 113.08 dB lower than the uniform array, 68.78 dB lower than the PSO [31] algorithm-optimized array, 50.63 dB lower than the CSO [19] algorithm optimized array, 23.98 dB lower than the POA [34]-optimized array, and 8 dB lower than the DO algorithm-optimized array, and the main lobe width has not been widened, still within the specified main lobe range either. It can also be seen that the uniform array, PSO [31] algorithm, CSO [19] algorithm, and POA [34] provide SLL peaks of −13.2360 dB, −18.7300 dB, −18.1537 dB, and −19.7400 dB, respectively. The DO algorithm can reduce the peak SLL to −20.1759 dB, while the CENDO algorithm can obtain a peak sidelobe level of −20.9227 dB, which is equivalent to a reduction of 7.6867 dB compared to the uniform array, 2.1927 dB compared to the PSO [31] algorithm, 2.7690 dB compared to the CSO [19] algorithm, 1.1827 dB compared to the POA [34] algorithm, and 0.7468 dB compared to the DO algorithm. From Table 8, the main lobe width obtained by the CENDO algorithm is slightly widened compared to other algorithms, but it does not exceed the designated main lobe range by 10°. However, for this array antenna model, the main purpose is to obtain deeper zero points and lower sidelobe levels to enhance anti-jamming capability. Without a doubt, the zero point obtained by using the CENDO algorithm is deeper, and the sidelobe level is also lower, which is crucial for the research and design of array antennas. Thus, in terms of optimizing array antenna performance, the CENDO algorithm outperforms the PSO [31] algorithm, CSO [19] algorithm, POA [34], and DO algorithm.

Experimental Simulation 5: This example shows how to use the DO algorithm and CENDO algorithm to optimize the positions of a 28-element linear array for SLL minimization and the formation of deep zero points. SLL reduction is desired in the regions *θ* = [0°, 84°] and *θ* = [96°, 180°], and deep nulls at *θ* = 55°, 57.5°, 60°, 120°, 122.5° and 125°. The population size and the maximum number of iterations are set to 30 and 1000, respectively.

**Figure 15:**
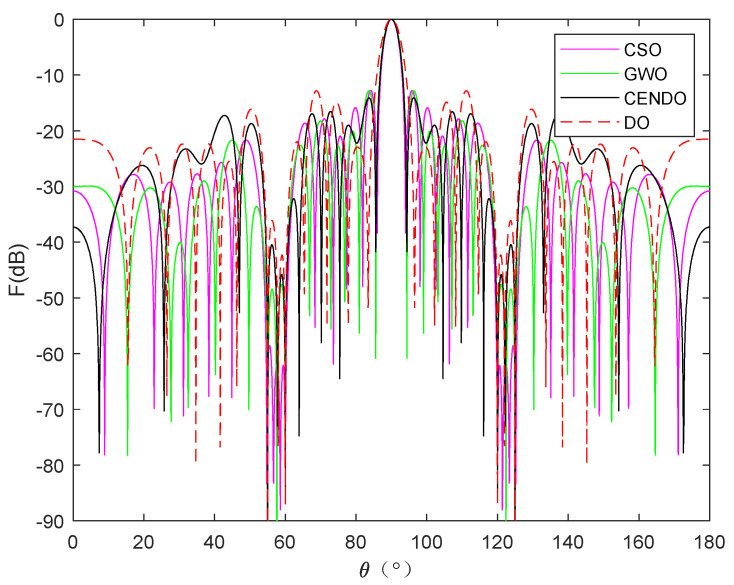
The array radiation patterns optimized by CSO [19], GWO [35], DO, and CENDO algorithms.

**Figure 16:**
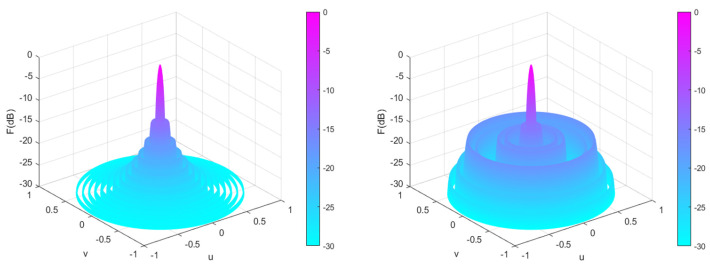
Unoptimized 3D radiograms (**left**) and 3D radiograms optimized by the CENDO algorithm (**right**).

Figure 15 shows the array patterns optimized by the CSO [19], GWO [35], DO, and CENDO algorithms. From Figure 15, it can be visually observed the directional pattern results of the array optimized by each algorithm. From the figure, it can be observed that the SLL optimized by the CENDO algorithm is the lowest, and its depth is lowest in the deepest of the six nulls in the specified direction. Therefore, the CENDO algorithm has a better ability to reduce array SLL and nulls compared to other comparative algorithms. The 3D radiograms (two-dimensional planes rotating 180° around the z-axis) before and after optimization are demonstrated by Figure 16. Table 9 provides the optimized positions of the array elements obtained by the CSO [19] algorithm, the GWO [35] algorithm, the DO algorithm, and the CENDO algorithm. Table 10 shows the peak SLL values, the nulls depth, and the main lobe width. From the characterization of the anti-jamming capability of the array antenna, it is very easy to see that the null depths provided by the CSO [19] algorithm are −65.37 dB, −62.15 dB, −83.69 dB, −83.69 dB, −62.15 dB, and −65.37 dB, where −83.69 dB is the deepest zero value it can obtain. GWO [35] algorithm gives −74.50 dB, −67.27 dB, −72.83 dB, −72.83 dB, −67.27 dB, and −74.50 dB zeros and the deepest zero-point value is −74.50 dB. Using the original DO algorithm, the zero values obtained are −90.11 dB, −44.26 dB, −87.17 dB, −87.17 dB, −44.26 dB, and −90.11 dB, respectively; the deepest zero value is −90.11 dB. However, the nulls depth generated by the CENDO algorithm are −97.25 dB, −51.65 dB, −86.57 dB, −86.57 dB, −51.65 dB, and −97.25 dB, respectively. Among them, the deepest zero point that can be obtained in the desired direction can reach as high as −97.25 dB (*θ* = 55° and *θ* = 125°), which is achieved through the CENDO algorithm. Comparing the results of the deepest zero point above, it is found that the nulls depth obtained by the array optimized using the CENDO algorithm is 13.56 dB lower than that optimized by the CSO [19] algorithm, 22.75 dB lower than that optimized by the GWO [35] algorithm, and 7.14 dB lower than that optimized by the original DO algorithm. It can also be seen that the peak SLL values provided by the CSO [19] algorithm, the GWO [35] algorithm, and the DO algorithm are −12.8606 dB, −12.8683 dB, and −12.8351 dB, respectively. The CENDO algorithm can reduce the peak SLL value to −14.1049 dB, which is equivalent to a decrease of 1.2443 dB compared to CSO [19] algorithm, 1.2366 dB compared to GWO [35] algorithm and 1.2698 dB compared to the original DO algorithm, and the main lobe is not widened either. So, the array antenna optimized using the CENDO algorithm is more resistant to interference than the other three algorithms. All in all, the CENDO algorithm is superior to the original DO algorithm, the CSO [19] algorithm, and the GWO [35] algorithm in the optimization of the antenna array pattern synthesis design.

## 5. Summary

Based on the original dandelion algorithm, this paper introduces an improved dandelion optimization algorithm called the chaos exchange nonlinear dandelion optimization algorithm. Additionally, it is used to synthesize non-equidistant linear arrays in the area of electromagnetism. To verify the effectiveness of the algorithm, the improved CENDO algorithm is tested and compared with the original DO algorithm, as well as the PSO, GSA, and PSOGSA algorithms on 10 benchmark functions, and conclusions are easily drawn: the CENDO algorithm shows a higher performance on these test functions. Then, simulation research is conducted on non-equidistant linear arrays, and the optimization results of the CENDO algorithm, DO algorithm, and other popular algorithms are compared. Through comparative analysis, using the CENDO algorithm for optimization can not only obtain array patterns with lower sidelobe levels and deeper nulls, but also the main lobe is basically not widened, and the electromagnetic energy radiated in the main lobe direction is also relatively concentrated. From this, we can conclude that the CENDO algorithm has superior optimization capabilities in array antenna synthesis compared to the DO algorithm and other popular algorithms. Overall, the CENDO algorithm is a successful improvement and can serve as an excellent candidate method in the field of array antenna synthesis. However, it also has certain shortcomings, one is that when increasing the number of antenna array elements as well as increasing the optimization objective, the optimization speed of the algorithm becomes slower compared to optimizing a simple array model and a simple optimization objective, and the optimization of complex non-equally spaced antenna arrays takes longer. Secondly, the scope of the algorithm to solve the problem may be limited, this paper only discusses the CENDO algorithm to solve the problem of non-equal spacing antenna array optimization, highlighting the advantages of the algorithm, while ignoring the study of other types of arrays, such as time-modulated array antennas, two-dimensional planar array antennas, or the number of hundreds of large-scale array antennas, If it is extended to more complex antenna array pattern optimization problems, the CENDO algorithm may not necessarily exhibit better optimization performance. Of course, these shortcomings will also become the focus of future research. In addition, we will no longer limit ourselves to theoretical simulation optimization of antenna models but move closer to engineering applications closer to the real world. According to the experimental simulation design of different antenna arrays conducted by our research institute, we can use the optimized optimal array index results to guide the actual antenna array design. By using EM software such as CST and HFSS to model and analyze the antenna array, we can then produce a matching real antenna array and use relevant electromagnetic testing tools to verify and test whether the sidelobe level and other related indicators reach the theoretical optimal value, forming a closed loop for comprehensive optimization design of antenna arrays. We strive to contribute to the field of antenna array research in electromagnetics.

## Figures and Tables

**Table 1:** Details of testing functions.

Function Name	Expression	Dimension(D)	Boundaries
**F1**	$f\left(x\right)=\sum_{i=1}^{D}{x_{i}}^{2}$	30	$-100\leq x_{i}\leq 100$
**F2**	$f\left(x\right)=\sum_{i=1}^{D}\left|x_{i}\right|+\prod_{i=1}^{D}\left|x_{i}\right|$	30	$-10\leq x_{i}\leq 10$
**F3**	$f\left(x\right)={\sum_{i=1}^{D}\left(\sum_{j=1}^{i}x_{j}\right)}^{2}$	30	$-100\leq x_{i}\leq 100$
**F4**	$\max\left\{\left|x_{i}\right|,1\leq i\leq D\right\}$	30	$-100\leq x_{i}\leq 100$
**F5**	$f\left(x\right)=\sum_{i=1}^{D-1}\left[100{\left(x_{i+1}-{x_{i}}^{2}\right)}^{2}+{\left(x_{i}-1\right)}^{2}\right]$	30	$-30\leq x_{i}\leq 30$
**F6**	$f\left(x\right)=\sum_{i=1}^{D}{\left(\left|x_{i}+0.5\right|\right)}^{2}$	30	$-100\leq x_{i}\leq 100$
**F7**	$f\left(x\right)=\sum_{i=1}^{D}{{\mathrm{ix}}_{i}}^{4}+\mathrm{rand}\left[0,1\right)$	30	$-1.28\leq x_{i}\leq 1.28$
**F9**	$f\left(x\right)=\sum_{i=1}^{D}\left[{x_{i}}^{2}-10\cos\left(2\pi x_{i}\right)+10\right]$	30	$-5.12\leq x_{i}\leq 5.12$
**F11**	$f\left(x\right)=\frac{1}{4000}\sum_{i=1}^{D}{x_{i}}^{2}-\prod_{i=1}^{D}\cos\left(\frac{x_{i}}{\sqrt{i}}\right)+1$	30	$-600\leq x_{i}\leq 600$
**F12**	$\begin{array}{l} f\left(x\right)=\frac{\pi }{D}\left\{10\sin\left(\pi y_{1}\right)\right.\\ \left.+\sum_{i=1}^{D-1}{\left(y_{i}-1\right)}^{2}\left[1+10\sin^{2}\left(\pi y_{i+1}\right)\right]+{\left(y_{D}-1\right)}^{2}\right\}\\ +\sum_{i=1}^{D}u\left(x_{i},10,100,4\right),y_{i}=1+\frac{x_{i}+1}{4}\\ u\left(x_{i},a,k,m\right)=\left\{\begin{array}{l} k{\left(x_{i}-a\right)}^{m},x_{i}>a\\ 0,-a<x_{i}<a\\ k{\left(-x_{i}-a\right)}^{m},x_{i}<-a \end{array}\right\} \end{array}$	30	$-50\leq x_{i}\leq 50$

**Table 2:** Comparison of the results of the test function in different cases.

Function Name		DO	CDO	EDO	NDO	CENDO
**F1**	best value	1.1350 × 10^−12^	1.5910 × 10^−12^	**3.0552 × 10^−49^**	**6.3899 × 10^−13^**	**1.1062 × 10^−58^**
	worst value	4.0869 × 10^−11^	**3.4068 × 10^−11^**	**7.0240 × 10^−38^**	**1.0308 × 10^−11^**	**4.0135 × 10^−46^**
	mean value	1.3561 × 10^−11^	**1.0879 × 10^−11^**	**2.0515 × 10^−39^**	**3.7274 × 10^−12^**	**1.7060 × 10^−47^**
	SD	8.7957 × 10^−12^	**7.2233 × 10^−12^**	**1.0035 × 10^−38^**	**2.3414 × 10^−12^**	**7.5989 × 10^−47^**
**F2**	best value	5.2944 × 10^−7^	6.9727 × 10^−7^	**2.5494 × 10^−30^**	**3.3167** × 10^−7^	**3.0504 × 10^−36^**
	worst value	8.5704 × 10^−6^	**5.5961 × 10** **^−6^**	**2.3854 × 10^−21^**	**2.7245 × 10** **^−6^**	**1.4932 × 10^−25^**
	mean value	1.8674 × 10^−6^	1.9004 × 10^−6^	**7.5076 × 10^−23^**	**9.8943 × 10^−7^**	**4.8151 × 10^−27^**
	SD	1.3876 × 10^−6^	**1.0431 × 10** **^−6^**	**3.6352 × 10^−22^**	**4.8112 × 10^−7^**	**2.3521 × 10^−26^**
**F3**	best value	6.1974 × 10^−4^	7.5102 × 10^−4^	**3.6225 × 10^−17^**	**5.5384 × 10^−5^**	**2.0115 × 10^−21^**
	worst value	1.7171 × 10^−2^	2.3690 × 10^−2^	**3.2378 × 10^−6^**	**3.9179 × 10^−3^**	**2.5321 × 10^−7^**
	mean value	6.3481 × 10^−3^	**4.8962 × 10^−3^**	**1.5462 × 10^−7^**	**6.5332 × 10^−4^**	**5.5930 × 10^−9^**
	SD	4.4766 × 10^−3^	**4.3306 × 10^−3^**	**6.2989 × 10^−7^**	**6.9211 × 10^−4^**	**3.5918 × 10^−8^**
**F4**	best value	1.0294 × 10^−3^	**8.8960 × 10^−4^**	**3.5817 × 10^−12^**	**3.6853 × 10^−4^**	**2.7001 × 10^−15^**
	worst value	1.3375 × 10^−2^	1.4937 × 10^−2^	**8.9236 × 10^−8^**	**4.7220 × 10^−3^**	**7.0016 × 10^−8^**
	mean value	4.7372 × 10^−3^	**3.7680 × 10^−3^**	**1.0218 × 10^−8^**	**1.7663 × 10^−3^**	**1.4556 × 10^−9^**
	SD	3.0685 × 10^−3^	**2.3212 × 10^−3^**	**2.0412 × 10^−8^**	**1.0332 × 10^−3^**	**9.8948 × 10^−9^**
**F5**	best value	23.7842	**23.4837**	**22.5691**	**21.9988**	**22.7700**
	worst value	94.7012	**29.2299**	**23.7477**	95.3098	**23.7119**
	mean value	25.4670	**24.0960**	**23.0311**	**25.0807**	**23.3024**
	SD	9.9919	**0.7644**	**0.1993**	10.1405	**2.1058 × 10^−1^**
**F6**	best value	2.5976 × 10^−8^	4.1893 × 10^−8^	**1.8420 × 10** **^−8^**	**2.5850 × 10^−8^**	**1.7011 × 10^−8^**
	worst value	3.0494 × 10^−7^	**2.4210 × 10^−7^**	**1.6911 × 10^−7^**	**2.0875 × 10^−7^**	**1.4545 × 10^−7^**
	mean value	1.1025 × 10^−7^	1.1706 × 10^−7^	**6.6178 × 10^−8^**	**7.4026 × 10^−8^**	**6.1266 × 10^−8^**
	SD	6.5961 × 10^−8^	**4.8672 × 10^−8^**	**3.3529 × 10^−8^**	**3.2753 × 10^−8^**	**3.0873 × 10^−8^**
**F7**	best value	7.4573 × 10^−4^	1.1608 × 10^−3^	**6.1817 × 10^−5^**	**2.6884 × 10^−4^**	**3.4343 × 10^−5^**
	worst value	5.8979 × 10^−3^	7.4474 × 10^−3^	**2.9432 × 10^−3^**	**5.1711 × 10^−3^**	**3.6334 × 10^−3^**
	mean value	2.7707 × 10^−3^	3.3089 × 10^−3^	**9.5340 × 10^−4^**	1.4736 × 10^−3^	**7.3110 × 10^−4^**
	SD	1.3803 × 10^−3^	1.4179 × 10^−3^	**6.9126 × 10^−4^**	**1.1688 × 10^−13^**	**5.9049 × 10^−4^**
**F9**	best value	6.1959 × 10^−12^	**3.6380 × 10^−12^**	**0**	**1.7621 × 10^−12^**	**0**
	worst value	38.8923	**38.8576**	**31.4955**	**29.8687**	49.8096
	mean value	8.0689	13.4624	**7.2478**	10.4583	13.5670
	SD	7.2886	9.8276	8.0668	**6.6158**	14.1309
**F11**	best value	2.1116 × 10^−11^	**1.1190 × 10^−11^**	**0**	**3.0228 × 10^−12^**	**0**
	worst value	7.0601 × 10^−2^	**5.6428 × 10^−2^**	1.0290 × 10^−1^	1.6368 × 10^−1^	**6.4158 × 10^−2^**
	mean value	1.6357 × 10^−2^	**1.2401 × 10^−2^**	**5.0190 × 10^−3^**	1.6687 × 10^−2^	**4.5923 × 10^−3^**
	SD	1.7744 × 10^−2^	**1.5054 × 10^−2^**	**1.5717 × 10^−2^**	0.3140 × 10^−1^	**1.1169 × 10^−2^**
**F12**	best value	2.4178 × 10^−9^	**2.0565 × 10^−9^**	**7.1240 × 10^−10^**	**1.0438 × 10^−9^**	**6.8597 × 10**
	worst value	1.3806 × 10^−8^	1.6267 × 10^−8^	**1.3358 × 10^−8^**	**9.0565 × 10^−9^**	**9.9351 × 10^−9^**
	mean value	6.6864 × 10^−9^	**6.4920 × 10^−9^**	**5.1745 × 10^−9^**	**4.3961 × 10^−9^**	**4.0078 × 10^−9^**
	SD	2.6938 × 10^−9^	2.7398 × 10^−9^	**2.5527 × 10^−9^**	**1.6232 × 10^−9^**	**2.1110 × 10^−9^**

**Table 3:** Comparison of test function results for different algorithms.

Function Name	Algorithm	Best Value	Mean Value
**F1**	**CENDO**	**1.1281 × 10^−48^**	**4.4247 × 10^−38^**
	**PSO**	8.75 × 10^−6^	2.83 × 10^−4^
	**GSA**	7.92 × 10^−17^	1.19 × 10^−16^
	**PSOGSA**	4.91 × 10^−19^	6.66 × 10^−19^
**F2**	**CENDO**	**3.4940 × 10^−29^**	**3.7062 × 10^−24^**
	**PSO**	7.07 × 10^−6^	5.50 × 10^−3^
	**GSA**	4.17 × 10^−8^	4.77 × 10^−8^
	**PSOGSA**	3.18 × 10^−9^	3.79 × 10^−9^
**F3**	**CENDO**	**2.5842 × 10^−11^**	**1.1836 × 10^−4^**
	**PSO**	1.91 × 10^3^	5.19 × 10^3^
	**GSA**	297.666	734.566
	**PSOGSA**	43.2038	409.936
**F4**	**CENDO**	**2.3070 × 10^−10^**	2.4021 × 10^−4^
	**PSO**	2.41 × 10^−8^	4.38 × 10^−7^
	**GSA**	9.72 × 10^−9^	1.47 × 10^−2^
	**PSOGSA**	2.96 × 10^−10^	**3.37 × 10^−10^**
**F5**	**CENDO**	24.0318	**24.5176**
	**PSO**	**15.5933**	201.665
	**GSA**	26.2566	35.0076
	**PSOGSA**	22.4221	56.2952
**F6**	**CENDO**	1.2355 × 10^−7^	5.5031 × 10^−7^
	**PSO**	4.51 × 10^0^	4.96 × 10^0^
	**GSA**	8.17 × 10^−17^	1.67 × 10^−16^
	**PSOGSA**	**5.76 × 10^−19^**	**7.40 × 10^−19^**
**F7**	**CENDO**	**6.3937 × 10^−4^**	**3.1563 × 10^−3^**
	**PSO**	1.05 × 10^−1^	2.60 × 10^−1^
	**GSA**	8.07 × 10^−2^	4.58 × 10^−1^
	**PSOGSA**	2.77 × 10^−2^	5.09 × 10^−2^
**F9**	**CENDO**	**0**	**20.4782**
	**PSO**	55.7182	72.9581
	**GSA**	24.1444	31.1185
	**PSOGSA**	19.1371	22.6777
**F11**	**CENDO**	**0**	9.6875 × 10^−3^
	**PSO**	9.96 × 10^−7^	5.43 × 10^−3^
	**GSA**	3.96 × 10^0^	6.98 × 10^0^
	**PSOGSA**	1.11 × 10^−16^	**1.48 × 10^−3^**
**F12**	**CENDO**	**1.8897 × 10^−8^**	**3.2607 × 10^−7^**
	**PSO**	1.06 × 10^−1^	2.29 × 10^0^
	**GSA**	1.06 × 10^−1^	1.95 × 10^−1^
	**PSOGSA**	6.43 × 10^0^	23.4 × 10^0^

**Table 4:** Comparison of array element positions, peak SLL, main lobe width, and directivity of four algorithms.

Methods	x1 (λ)	x2 (λ)	x3 (λ)	x4 (λ)	x5 (λ)	Peak SLL (dB)	Main Lobe Width (deg)	Directivity (dB)
PSO [31]	0.2515	0.5550	1.0650	1.5000	2.1100	−17.41	27.2	9.7306
QPM [31]	0.2200	0.6250	1.1000	1.6100	2.2100	−17.09	25.8	9.9120
**DO**	**0.2076**	**0.6475**	**1.1072**	**1.6776**	**2.3411**	**−19.05**	**25.0**	**10.0871**
**CENDO**	**0.2063**	**0.6487**	**1.1080**	**1.6788**	**2.3468**	**−19.14**	**25.0**	**10.0930**

**Table 5:** The 16-element LAAs with optimized element positions, peak SLL, main lobe width, and directivity are shown using different algorithms.

Methods	Optimized Element Positions (λ)	Peak SLL (dB)	Main Lobe Width (deg)	Directivity (dB)
Uniform array	0.2500, 0.7500, 1.2500, 1.7500 2.2500, 2.7500, 3.2500, 3.7500	−13.1476	14.4	12.0412
PSO [32]	0.2500, 0.5311, 1.0128, 1.3930 1.8738, 2.3329, 2.9893, 3.7500	−21.3693	17.4	11.8379
PSOGSA [32]	0.2500, 0.5495, 1.0230, 1.3560 1.8561, 2.3358, 2.9783, 3.7500	−21.8484	17.4	11.8258
WOA [32]	0.2500, 0.6485, 1.0456, 1.3751 1.9467, 2.4634, 3.0076, 3.7500	−19.1546	16.6	11.8220
GOA [32]	0.2500, 0.5802, 1.1274, 1.3493 1.9119, 2.3129, 3.0208, 3.7500	−19.9808	17.0	11.8185
SSA [32]	0.2500, 0.5331, 1.0118, 1.3453 1.8495, 2.3404, 2.9835, 3.7500	−22.0177	17.6	11.8232
MSSA [32]	0.2500, 0.5226, 1.0038, 1.3486 1.8518, 2.3447, 2.9948, 3.7500	−22.6768	17.6	11.8312
**DO**	**0.2500, 0.5138, 1.0025, 1.3456** **1.8454, 2.3264, 2.9886, 3.7500**	**−22.8766**	**17.6**	**11.8228**
**CENDO**	**0.2500, 0.5127, 1.0011, 1.3461** **1.8456, 2.3274, 2.9885, 3.7500**	**−22.9377**	**17.6**	**11.8229**

**Table 6:** The optimized positions, peak SLL, main lobe width, and directivity of a 28-element array.

Methods	Optimized Element Positions (λ)							Peak SLL (dB)	Main Lobe Width (deg)	Directivity (dB)
Uniform array	0.2500 3.7500	0.7500 4.2500	1.2500 4.7500	1.7500 5.2500	2.2500 5.7500	2.7500 6.2500	3.2500 6.7500	−13.23	8.2	14.4715
PSO [19]	0.3270 3.4450	0.2089 4.3046	0.5771 4.8928	1.5145 5.1472	2.1417 5.9070	2.3939 6.4275	2.8792 6.9999	−17.09	8.6	13.6832
CSO [19]	0.2437 3.2657	0.6445 3.8500	1.0230 4.4726	1.5095 5.1068	1.8444 5.8367	2.3974 6.5065	2.8835 6.9999	−20.04	9.0	14.3526
RRA [33]	0.2236 3.2419	0.6239 3.8255	1.0019 4.4474	1.4878 5.0808	1.8223 5.8098	2.3747 6.4788	2.8602 6.9708	−20.37	9.0	14.3379
**DO**	**0.2022** **2.7729**	**0.4289** **3.1010**	**0.8344** **3.6891**	**1.1749** **4.0717**	**1.5251** **4.7160**	**1.9593** **5.3298**	**2.3173** **5.8249**	**−20.39**	**11.2**	**13.6416**
**CENDO**	**0.2352** **3.2331**	**0.6405** **3.6861**	**1.0524** **4.2620**	**1.4659** **4.9108**	**1.8690** **5.6723**	**2.2926** **6.3770**	**2.8156** **6.9822**	**−20.60**	**9.4**	**14.4040**

**Table 7:** A 32-element LAA with optimized element positions, peak SLL, and nulls depth.

Algorithms	Optimized Element Positions (λ)							Peak SLL (dB)	Nulls Depth (dB)
Uniform array	0.2500 3.7500 7.2500	0.7500 4.2500 7.7500	1.2500 4.7500	1.7500 5.2500	2.2500 5.7500	2.7500 6.2500	3.2500 6.7500	−13.2360	−17.82
PSO [31]	0.2650 3.0550 7.0500	0.6850 3.4300 7.7550	1.1750 3.9000	1.5550 4.3800	1.9850 4.9500	2.3300 5.5500	2.6650 6.2400	−18.7300	−62.12
CSO [19]	0.2883 3.1362 7.0412	0.6830 3.4848 7.7500	1.1929 3.9538	1.5199 4.3822	1.9768 4.9252	2.3247 5.4817	2.6886 6.2091	−18.1537	−80.27
POA [34]	0.3049 2.9572 6.7426	0.5115 3.4099 7.4663	1.0215 3.6838	1.3986 4.1534	1.8307 4.4520	2.1033 5.1762	2.5361 5.9173	−19.7400	−106.92
**DO**	**0.2996** **2.8647** **7.1681**	**0.5609** **3.2644** **7.9981**	**0.9371** **3.6225**	**1.3817** **4.0510**	**1.6445** **4.6348**	**2.1112** **5.3695**	**2.3166** **6.2077**	**−20.1759**	**−122.9**
**CENDO**	**0.3138** **2.8447** **6.8829**	**0.4052** **3.0164** **7.6252**	**0.8996** **3.5188**	**1.2376** **3.8911**	**1.5695** **4.3711**	**2.1239** **5.0393**	**2.2640** **5.7357**	**−20.9227**	**−130.9**

**Table 8:** Main lobe width obtained using different algorithms.

**Algorithms**	Uniform Array	PSO [31]	CSO [19]	POA [34]	**DO**	**CENDO**
**Main lobe width (deg)**	7.2	8.4	8.4	9.0	**9.8**	**10.0**

**Table 9:** The positions of the elements of the 28-element array obtained are optimized using four algorithms.

Algorithms	Optimized Element Positions (λ)						
CSO [19]	0.2720 3.7693	0.7547 4.2222	1.1399 4.8991	1.7065 5.4061	2.3287 5.7389	2.8675 6.1564	3.3536 6.7173
GWO [35]	0.2298 3.4867	0.7266 3.7652	1.1376 4.3933	1.7300 4.9039	2.1831 5.3422	2.6710 5.7610	3.0255 6.3378
**DO**	**0.2000** **2.5315**	**0.5643** **3.1079**	**0.7496** **3.5366**	**1.1504** **3.9885**	**1.3564** **4.0472**	**1.8027** **4.6043**	**2.1642** **6.9910**
**CENDO**	**0.3209** **3.5615**	**0.9922** **3.7629**	**1.3212** **4.0276**	**1.8889** **4.5805**	**2.3351** **5.5499**	**2.7596** **6.3469**	**2.9895** **7.0000**

**Table 10:** Comparison of CENDO-based results with other algorithms for the 28-element array design.

**Algorithms**	CSO [19]	GWO [35]	**DO**	**CENDO**
**Peak SLL (dB)**	−12.8606	−12.8683	**−12.8351**	**−14.1049**
**Nulls depth (dB)**				
**Θ = 55°**	−65.37	−74.50	**−90.11**	**−97.25**
**Θ = 57.5°**	−62.15	−67.27	**−44.26**	**−51.65**
**Θ = 60°**	−83.69	−72.83	**−87.17**	**−86.57**
**Θ = 120°**	−83.69	−72.83	**−87.17**	**−86.57**
**Θ = 122.5°**	−62.15	−67.27	**−44.26**	**−51.65**
**Θ = 125°**	−65.37	−74.50	**−90.11**	**−97.25**
**Main lobe width (deg)**	8.0	8.8	**13.2**	**8.8**

## Data Availability

The original contributions presented in this study are included in the article. Further inquiries can be directed at the author.
